# Targeted exon sequencing fails to identify rare coding variants with large effect in rheumatoid arthritis

**DOI:** 10.1186/s13075-014-0447-7

**Published:** 2014-09-30

**Authors:** So-Young Bang, Young-Ji Na, Kwangwoo Kim, Young Bin Joo, Youngho Park, Jaemoon Lee, Sun-Young Lee, Adnan A Ansari, Junghee Jung, Hwanseok Rhee, Jong-Young Lee, Bok-Ghee Han, Sung-Min Ahn, Sungho Won, Hye-Soon Lee, Sang-Cheol Bae

**Affiliations:** Department of Rheumatology, Hanyang University Hospital for Rheumatic Diseases, 220 Wangsimni-ro, Seoul, 133-792 Republic of Korea; Department of Applied Statistics, Chung-Ang University, 29 Heukseong-no, Seoul, 156-755 Republic of Korea; Department of Oncology, Asan Medical Center, 88 Olympic-ro 43-gil, Seoul, 138-736 Republic of Korea; Bioinfomatics Center, Macrogen Inc., 60-24, Gasan-dong, Seoul, 153-023 Republic of Korea; Center for Genome Science, Korea National Institute of Health, Osong Health Technology, 187 Osongsaengmyeong 2-ro, Chungcheongbuk-do, 363-700 Republic of Korea; Department of Biomedical Informatics, Asan Medical Center, 88 Olympic-ro 43-gil, Seoul, 138-736 Republic of Korea; Public Health Science, Graduate School of Public Health, Seoul National University, 1 Kwanak-ro Kwanak-gu, Seoul, 151-742 Republic of Korea

## Abstract

**Introduction:**

Although it has been suggested that rare coding variants could explain the substantial missing heritability, very few sequencing studies have been performed in rheumatoid arthritis (RA). We aimed to identify novel functional variants with rare to low frequency using targeted exon sequencing of RA in Korea.

**Methods:**

We analyzed targeted exon sequencing data of 398 genes selected from a multifaceted approach in Korean RA patients (*n* = 1,217) and controls (*n* = 717). We conducted a single-marker association test and a gene-based analysis of rare variants. For meta-analysis or enrichment tests, we also used ethnically matched independent samples of Korean genome-wide association studies (GWAS) (*n* = 4,799) or immunochip data (*n* = 4,722).

**Results:**

After stringent quality control, we analyzed 10,588 variants of 398 genes from 1,934 Korean RA case controls. We identified 13 nonsynonymous variants with nominal association in single-variant association tests. In a meta-analysis, we did not find any novel variant with genome-wide significance for RA risk. Using a gene-based approach, we identified 17 genes with nominal burden signals. Among them, *VSTM1* showed the greatest association with RA (*P* = 7.80 × 10^−4^). In the enrichment test using Korean GWAS, although the significant signal appeared to be driven by total genic variants, we found no evidence for enriched association of coding variants only with RA.

**Conclusions:**

We were unable to identify rare coding variants with large effect to explain the missing heritability for RA in the current targeted resequencing study. Our study raises skepticism about exon sequencing of targeted genes for complex diseases like RA.

**Electronic supplementary material:**

The online version of this article (doi:10.1186/s13075-014-0447-7) contains supplementary material, which is available to authorized users.

## Introduction

Rheumatoid arthritis (RA (MIM 180300)) is a complex autoimmune disorder that results from both genetic and environmental risk factors [1,2]. Strong evidence regarding the existence of a genetic predisposition for RA has been supported by several familial studies including twin studies, in which the heritability of RA has been estimated to be approximately 65% [1,2].

Nearly 60 RA risk loci were identified in several large studies including genome-wide association studies (GWAS) [3–6] and immunochip (iCHIP) [7,8] arrays using common single nucleotide variants (SNVs). The largest genetic contribution effect size has been identified for the major histocompatibility complex (MHC) locus with evidence for three independent association signals on *HLA-B, HLA-DRB1*, and *HLA-DPB1* affecting five amino acid positions [9,10]. The total variance of the MHC region explained 13.03% of the RA risk [11]. The other non-MHC genes identified were primarily immune pathway genes, though their effect sizes were quite modest. To date, the known RA risk loci can explain only about 25% of the total genetic heritability [12].

It has been suggested that rare or low-frequency variants could explain the substantial unexplained heritability of many complex diseases, most of which were not fully captured using the previous conventional genotyping technology. Recently, Stahl *et al.* [11] inferred a highly polygenic model that attempted to explain the missing heritability of RA. In this model, it was suggested that a small number of rare variants with large effect sizes may contribute to heritability in addition to hundreds of common variants.

New genomic technologies, including next-generation sequencing (NGS), can provide a new approach for identification of rare variants. With advances in NGS technology, the role of rare or low-frequency variants in many complex diseases like RA can be investigated to better characterize the genetic architecture of the disease. Recently, several sequencing studies that have investigated common autoimmune diseases have shown that rare variants within genes containing common variants are associated with complex diseases [13–16]. For RA, Diogo *et al.* [17] performed deep exon sequencing of 25 biological candidate genes discovered by GWASs in 500 RA cases and 650 controls of European ancestry and subsequent dense genotyping in larger samples, in which they found accumulation of a few rare nonsynonymous variants with nominal significance instead of variants with large effect of genome-wide significance.

Here, we aimed to identify novel functional variants with rare to low frequency using targeted exon sequencing in Korean RA, which dealt with hundreds of selected genes that were related to RA in various aspects such as previous identified genes from GWASs and immunochip data, literature reviews, and related pathways.

## Materials and methods

### Patients and controls

A total of 1,252 RA cases were enrolled from the BAE cohort of Hanyang University Hospital for Rheumatic Diseases and satisfied the American College of Rheumatology 1987 classification criteria [18] for RA. The ethnically matched 745 healthy controls, excluding those with a personal or familial history of any autoimmune disease, were recruited at the same institute. Informed consent was obtained from all individuals via a questionnaire at the time of enrollment, when clinical information was also collected. The study was approved by the institutional review board of Hanyang University (HYG-11-015-1).

### Targeted gene selection

We selected candidate genes using a comprehensive approach that included previous genetic and biological research, pathway databases, text-mining analysis, and animal-model databases (Figure S1 in Additional file 1). Of the non-MHC candidate genes, we included (a) 106 known RA risk loci identified via literature review [3,4,6,9,19], (b) 519 genes associated with RA in our Korean iChip dataset [8], (c) 155 genes shared by both RA and systemic lupus erythematosus (SLE) in our previous Korean GWAS datasets [4,20], (d) 18 genes in RA-related pathways, (e) 65 genes identified via text mining using GRAIL from recent GWAS data [3,4], and (f) 8 human homologs of mouse genes that induced an RA-like phenotype from the Mouse Genome Database (MGD) [21]. Altogether, 666 designable target genes were selected for exon sequencing.

### Exon sequencing

We enriched the target exons with Agilent’s SureSelect capture kit (target region = 1.36 Mb) and performed high-throughput paired-end sequencing using a HiSeq2000 (Agilent Technologies, Santa Clara, CA, USA). The sequencing reads were mapped to the human reference genome, where the reference sequence was UCSC assembly hg19 (NCBI build 37.1) using Burrows-Wheeler Aligner (BWA) software [22]. We then applied programs packaged in Picard-tools in order to convert the previous SAM file into a format that was sorted by mapping coordinates and to remove PCR duplicates. We created another SAM file that included only reads that uniquely mapped to the reference genome, and transformed this into a BAM file using Samtools [23]. Those variants are annotated by ANNOVAR (Figure S2 in Additional file 1).

We then performed stringent quality control for 666 target gene by which we selected only high coverage genes that were sequenced coding-region based on the public database (db). We obtained an initial dataset with 50,247 variants from 666 targeted genes. We filtered the original genotype matrix by single nucleotide polymorphism (SNP) quality and depth coverage. The filtered genotype data contains genotype calls satisfying with practical guidance in rare variants analysis of complex trait association studies [24]; coding sites sequenced with >20× coverage and quality score >30 in at least 80% of cases and controls in the public database (db) or in the designed targeting genes. As a result, a total of 398 genes (mean 92.0% coverage of the captured exon) in 1,997 individuals were used in the subsequent variant-calling analysis (Table S1 in Additional file 1). When we called the variants if SNVs had minimal depth coverage >20× and a quality score >30 in more than 80% of the subjects sequenced, a total of 12,916 variants within 398 genes were identified. We then eliminated SNVs that had insufficient call rates (<90%) in cases and controls, Hardy-Weinberg disequilibrium with *P* <0.01 in controls, and also eliminated samples that had insufficient call rates (<90%). We finally analyzed the 10,588 exonic variants of 1,934 samples for further single-variant association test, gene-based test, and pathway-based association enrichment test (Table 1, Table S2 in Additional file 1). There was no evidence of skewed genotyping between cases and controls in the principle component (PC) analysis (Figure S3 in Additional file 1).

**Table 1:** **Characteristics of RA patients and controls included in targeted exon sequencing**

	**RA cases (n = 1,217)** ^*^	**Controls (n = 717)** ^*^
Age of onset (mean ± SD years)	41.9 ± 12.8	35.1 ± 10.7
Disease duration (mean ± SD years)	9.9 ± 11.1	-
Female (%)	87.4	85.3
Rheumatoid factor (%)	97.8	-
Anti-cyclic citrullinated peptide autoantibodies (%)	98.1	-

^*^Cryptic relatedness with duplicate or first-degree relatives using KING software, outlier (deviating >8 SEM on any of the five principal components), or samples with less than 80% of the data sequenced were removed. A total of 1,934 samples were included for further analysis. SD, standard deviation; SEM, standard error of the mean.

### Statistical analysis

To analyze for single-marker association with RA in targeted exon sequencing data, odds ratios (OR) and *P* values were calculated using PLINK v1.07 software [25] with adjustments for the top 10 PCs in logistic regression. Fisher’s exact tests were also used for association tests of each rare variant.

For a meta-analysis (3,580 RA cases and 7,938 controls) using a Korean RA GWAS [4] and iCHIP [8] data generated from ethnically matched independent sample collections in addition to the current NGS results, we applied several quality-control filters on Korean RA GWAS (n = 4,799) and iCHIP (n = 4,722) data to select high-quality SNVs (minor allele frequency (MAF) ≥1%, *P* value of Hardy-Weinberg equilibrium (HWE) <10^−4^ and call rate >95% in cases and controls).

For an enrichment test for exonic SNPs in 77 newly identified genes (*P* <0.05 in a single-variant test of sequencing data), we imputed common and low-frequency variants (MAF >0.5%) from the Korean GWAS data (800 RA cases and 3,999 controls) by ShapeIt and IMPUTE2 with the 1000 Genomes Phase I reference panel. We performed logistic regressions for 1,000-times permuted phenotypes with the top 10 PCs as covariates by PLINK. Then, the numbers of genic, exonic, nonsynonymous, or synonymous variants reaching the *P*_threshold_ <0.05 between observed and permuted data were compared by using a Fisher’s exact test.

In a gene-based analysis of rare coding variants (MAF <5%), we performed both nonburden testing (optimal sequence kernel association test (SKAT-O)) [26] and burden testing (SCORE-seq) [27,28]. Weighted analysis was performed for rare nonsynonymous variants using SIFT [29], PolyPhen2 [30], and CAROL [31] scores. Statistical significance was determined by using 1,000,000 case-control permutations.

In pathway-based enrichment test of NGS data, we generated 1,000 permuted phenotype sets and their disease association *P* values for each SNV by logistic regression with adjustment for the top 10 PCs. To eliminate linkage disequilibrium (LD)-derived enrichment bias, we clumped the set of SNVs (*r*^*2*^ <0.4) in order of statistical significance. Then, we compared the number of SNV with *P* <*P*_threshold_ between permuted datasets and empirical datasets by Fisher’s exact tests. Genes in each functional pathway were obtained from Ontology and KEGG.

### Ethics approval

Ethics approval was granted by the institutional ethics committee of Hanyang University in the Republic of Korea.

Patient and control consents were obtained.

## Results

After stringent quality control on the 666 targeted genes (Figure S1 in Additional file 1) as well as the sequenced samples (see Materials and Methods), we obtained a final dataset for analysis that consisted of 10,588 exonic variants from 398 genes in 1,217 RA cases (age = 41.9 ± 12.8 (mean age ± standard deviation (SD)); female = 87.4%) and 717 controls (age = 35.1 ± 10.7 (mean age ± SD); female = 85.3%) (Table 1).

The majority of SNVs were rare (90.6% with MAF <5%), and 6,605 SNVs were novel which were not identified in the 1000 Genome Project dataset (Figure S4 in Additional file 1). The transition/transversion (Ti/Tv) rate was 2.93 in RA cases and 2.92 in controls, which indicates good quality control based on expected human mutation types (Table S2 in Additional file 1). We note that a high concordance rate was observed between genotype calls from sequencing versus other genotyping methods such as GWAS and iCHIP by non-reference sensitivity [32] and non-reference discrepancy rate [33] (Table S3 in Additional file 1). In addition, validation using a TaqMan assay for a selected 37 SNVs showed high concordance rates with sequencing data (99.3%).

**Figure 1:**
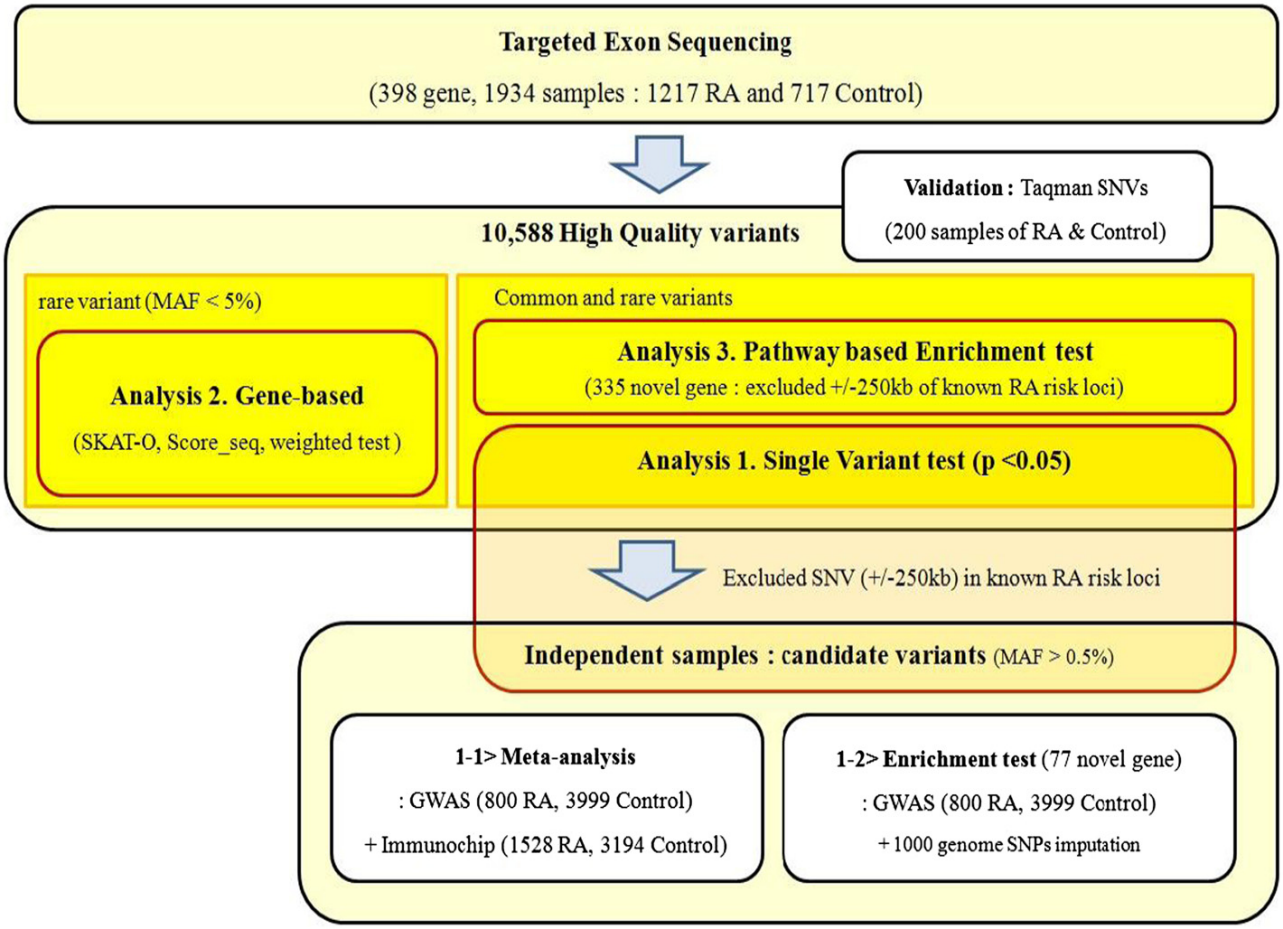
**Description of the study design.** We analyzed data from protein-coding variants within targeted genes using three different strategies for analysis.

We used three different strategies for analysis of variants that passed the stringent quality control: (1) single-variant association test, (2) gene-based test for rare variants of which MAF are less than 0.05, and (3) pathway-based association enrichment test (Figure 1). Regarding the single-variant association test, we further perform a meta-analysis using the current NGS data and previous GWAS/iCHIP data from independent Koreans, and evaluate that exonic SNPs in the genes associated with RA in the NGS data are enriched for RA association in imputed GWAS dataset.

In the first single-variant association analysis using 10,588 SNVs in 1,217 cases and 717 controls, we identified thirteen nonsynonymous variants with *P* <0.01, none of which reached the significance threshold after Bonferroni correction (Table S4 in Additional file 1). To compensate for the limited power that may have resulted in a lack of significant associations, we performed a meta-analysis for 108 coding SNVs (located in 89 genes) that were associated with a *P* value less than 0.05 in the single-variant association test. We used two independent Korean genomic data in the meta-analysis with NGS data; one was Korean RA GWAS dataset [4] (n = 1,099) combined with independent Korean control data (n = 3,700) genotyped by Illumina HumanOmni1-Quad BeadChip, and the other was Korean iCHIP data from 4,722 independent case-control subjects [8].

We focused on novel genes in the meta-analysis dataset of 3,580 RA cases and 7,938 controls, excluding any known GWAS or iCHIP signals. However, neither common nor low-frequency novel variants achieved genome-wide significance (*P* <1.0 × 10^−8^), with an SNV (rs1088680) within *PRKCH* showing the strongest association (*P* = 3.16 × 10^−5^) (Table S5 in Additional file 1).

**Figure 2:**
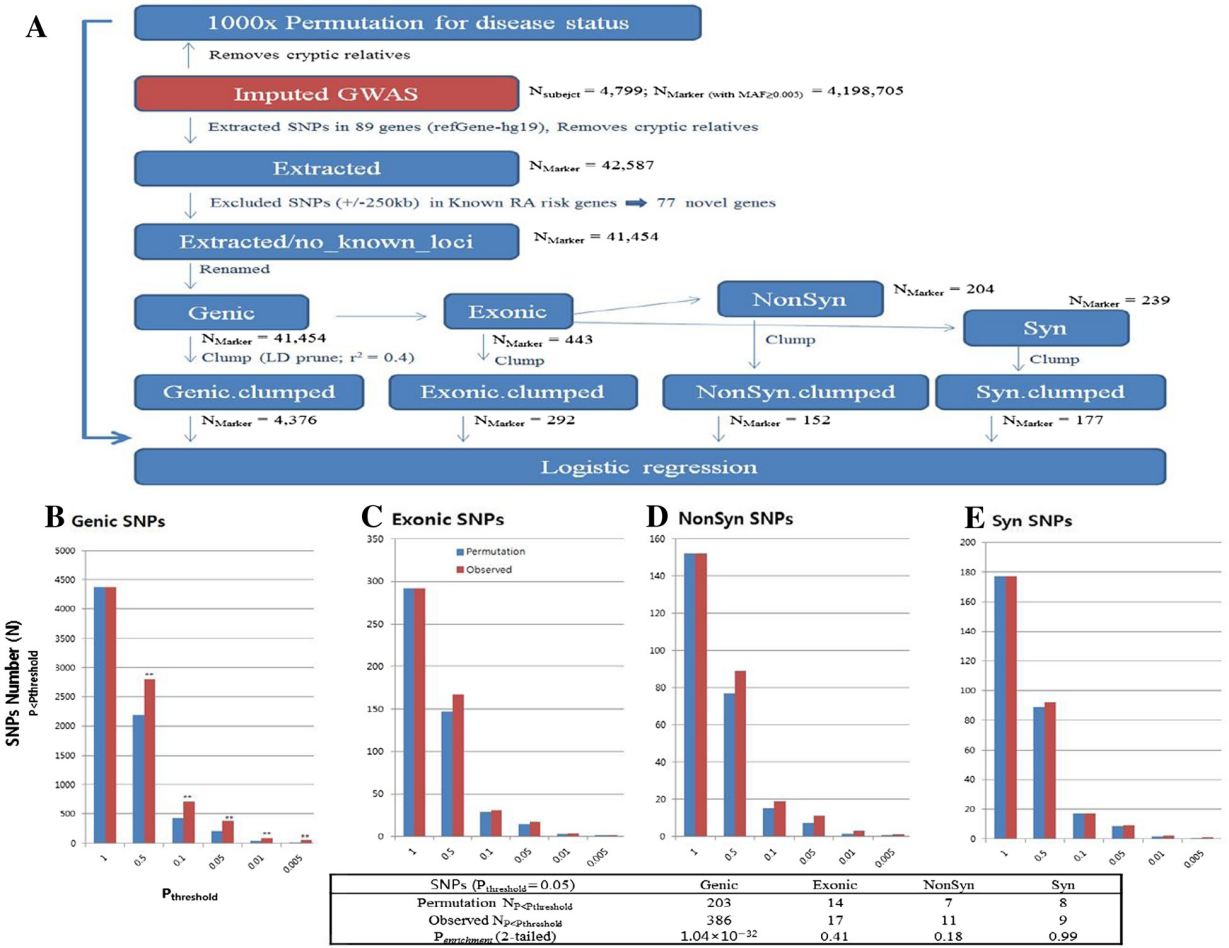
**Enrichment analysis of 77 novel genes with nominal signal on exon sequencing in the GWAS dataset. (A)** We performed logistic regressions including 10 principal components using 1,000-times permuted phenotypes. The numbers of **(B)** genic, **(C)** exonic, **(D)** nonsynonymous (NonSyn), and **(E)** synonymous (Syn) variants reaching the *P* <0.05 threshold following 1,000 permutations are shown. Significant enrichment of SNVs using the *P* <0.05 threshold was assessed using Fisher’s exact tests (^**^ *P* <0.01). These enrichment signals were driven by each group (*P*_*enrichment* (genic)_ = 1.04 × 10^−32^, *P*_enrichment (exon)_ = 0.41, *P*_enrichment (NonSyn)_ = 0.18, and *P*_*enrichment* (Syn)_ = 0.98 at *P*_*threshold*_ = 0.05).

Next, in an attempt to investigate whether an aggregate effect of 89 risk genes identified in NGS (*P* <0.05) exists or not, we performed an enrichment analysis for RA associations in all risk genes excluding 12 known RA risk loci using an independent dataset from our GWAS. Association of 41,454 SNVs within the 77 genes in the imputed Korean GWAS data was compared with the results after 1,000 case-control permutations by Fisher’s exact tests (Figure 2). To eliminate LD-derived bias, we clumped the set of independent SNVs (*r*^*2*^ <0.4).

Although the significant signal appeared to be driven by 4,376 genic variants (observed N_P<*P*threshold_/N_total_ = 386/4376; *P*_enrichment (genic)_ = 1.04 × 10^−32^), we found no evidence for enriched association at coding variants only (exon (observed N_P<*P*threshold_/N_total_ = 17/292; *P*_enrichment_ = 0.41 at *P*_threshold_ = 0.05), nonsynonymous (11/152; *P*_enrichment_ = 0.18), and synonymous (9/177; *P*_enrichment_ = 0.99)) (Figure 2).

**Figure 3:**
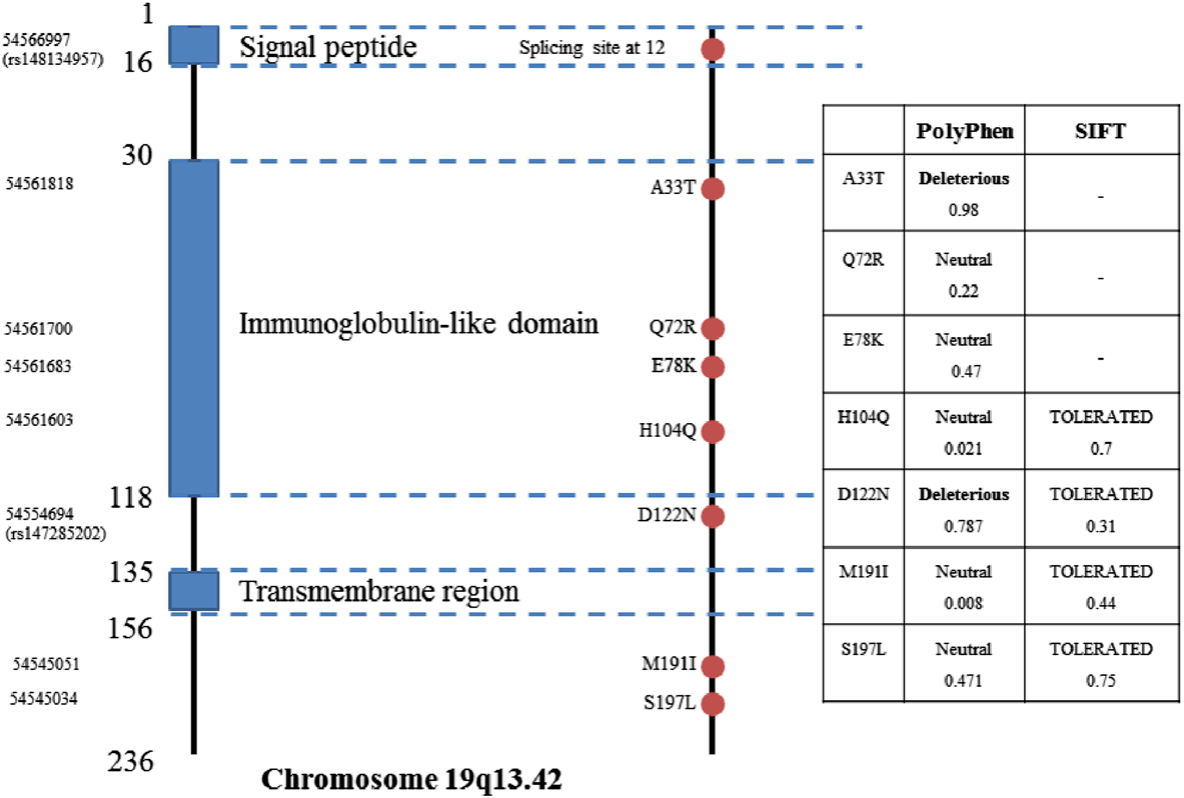
**Rare variants of** ***VSTM1*** **identified by exon sequencing.** The seven nonsynonymous variants of *VSTM1* that were validated by the Sanger sequencing method were primarily singletons driven from controls except for A33T (RA (n = 3), control (n = 5)). The four nonsynonymous variants of *VSTM1* lie within the coding regions of the immunoglobulin-like domain.

In the second analysis, we performed gene-based analysis of rare coding variant (MAF <5%) using nonburden tests (optimal sequence kernel association test (SKAT-O)) [26], burden tests (SCORE-seq) [28], and weighted tests with SIFT [29], PolyPhen2 [30], and CAROL [31] scores for the functional effects of the variants. A total of 17 genes had a nominal burden signal of association (*P* <0.05), most of which had two or more nonsynonymous rare variants, although they did not reach the threshold for significance after Bonferroni correction (*P* <1.2 × 10^−4^) (Table 2, Figure S5 in Additional file 1). For *VSTM1*, a top gene driven by the gene-based test, we further validated eight rare variants in *VSTM1* using Sanger sequencing with the same samples that were heterozygous for any of those variants in the initial sequencing stage; 29 of 31 samples were validated as heterozygous (false-positive rate = 6.45% (2/31)). The following analysis using only these validated samples showed that a set of the validated seven nonsynonymous variants of *VSTM1* conferred a protective role in RA (*P* = 4.55 × 10^−3^ in SKAT-O, *P* = 7.80 × 10^−4^ in SCORE-seq). The four variants of *VSTM1* were primarily within the immunoglobulin-like domain among the coding regions. The two variants, A33T at the domain and D122N closed to the domain, were thought to be deleterious variants by PolyPhen2, suggesting that these may have a functionally protective role in RA (Figure 3).

**Table 2:** **Gene-based tests of rare nonsynonymous variants in RA**

**GENE**	**Chr**	**Gene-based test** ^*^		
		**N**_**marker**_	**SKAT-O (nonburden test)** ***P*** **value**	**SCORE_seq (burden test)**
				***P*** **value**
*VSTM1*	19	7	4.55 × 10^−3^	7.80 × 10^−4^
*KPRP*	1	8	6.38 × 10^−3^	6.51× 10^−3^
*C6orf99*	6	5	0.05155	0.01669
*PARD3*	10	21	0.14496	0.01887
*PYGL*	14	10	0.02287	0.02057
*ARHGAP26*	5	9	0.01939	0.02114
*NCF2*	1	8	0.02574	0.02159
*CCR6*	6	7	0.01131	0.02776
*TRAF6*	11	8	0.04621	0.02876
*GRIN2B*	12	6	0.20806	0.02966
*SNTB1*	8	6	0.00987	0.03395
*PTCD3*	2	11	0.14462	0.03772
*CA8*	8	3	0.17982	0.03792
*NRXN3*	14	8	0.12536	0.03905
*CPEB4*	5	7	0.05104	0.04010
*CTNNA3*	10	16	0.10312	0.04079
*KRT24*	17	5	0.02866	0.04837

^*^We defined rare nonsynonymous variants as MAF <5% in both cases and controls. We selected 347 genes with two or more rare nonsynonymous variants for gene-based tests. RA, rheumatoid arthritis; Chr, chromosome; N_marker_, number of rare nonsynonymous variants for each gene.

In the third analysis, we conducted a pathway enrichment analysis of coding variants (nonsynonymous and synonymous) within 335 novel genes using Ontology and KEGG, which were obtained after excluding the known RA risk loci (+/-250 kb) from the initial 398 genes in NGS. In this analysis of both common and rare variants, we observed weak but significant evidence of overall enrichment of coding SNVs (*P*_*enrichment* (nonsynonymous)_ = 8.55 × 10^-4^ and *P*_*enrichment* (synonymous)_ = 0.166 at *P*_threshold_ = 0.05) (data not shown) for the 335 genes. However, in the analysis for each pathway, we did not identify any pathway in which a significant enrichment for coding variants for RA existed at *P*_threshold_ = 0.05.

## Discussion

This study does not support our hypothesis that the substantial proportion of missing heritability for RA can be inferred from rare coding variants. Among the 10,588 candidate variants of 398 genes analyzed in a cohort of 1,217 RA cases and 717 controls, 13 single nonsynonymous variants showed only nominal significance with a *P* value less than 0.01. Several genes found in the gene-based analysis also showed only weak association with RA.

Strikingly, this lack of a significant effect is consistent with that observed by Diogo *et al*. [17] in Caucasian population, in which most of the 25 candidate genes subjected to deep exon sequencing did not harbor rare coding variants contributing to risk of RA despite some evidence of accumulation of rare missense variants in gene-based test. Although 16 genes were overlapped between our Korean study and the Caucasian study [17], we could not find any significant single coding variant or gene that was shared in both ethnic groups. Taken together, rare functional variants may have very weak contribution to development of RA.

Besides, recent large-scale sequencing studies of six common autoimmune diseases (autoimmune thyroid disease, Crohn’s disease, celiac disease, psoriasis, multiple sclerosis, and type 1 diabetes) showed a negligible impact of rare autoimmune-locus coding variants on unexplained heritability (<3%) [34].

A possible reason for this negative finding may be the limited number of targeted genes that were sequenced, which were 398 genes in the current study. Rare coding variants of the remaining genes across whole genome could participate in the missing genetic contribution to RA. The other potential reason may be genetic heterogeneity for RA, in which each individual or small subset of RA may have their particular rare causal variants. The current study revealed a lot of examples in which a specific rare variant was observed in only one or two individuals among 1,934 subjects. To discover these lots of ‘private’ causal variants that are susceptible only in small subsets of RA patients, deep sequencing of whole exome from very large RA cases, approximately thousands of individuals, would be required.

*VSTM1*, a top signal gene driven by a gene-based test, is a glycoprotein primarily expressed in immune tissues, which can promote the differentiation and activation of Th17 cells [35]. In addition, two variants of *VSTM1* (A33T, D122N) found in the current study were deleterious variants by PolyPhen2. Therefore, it is certainly worth replicating these variants or performing deep sequencing of the entire *VSTM1* gene from an independent larger population, especially in Caucasians.

There are several limitations of this study. First, we performed a targeted exon sequencing study, which tends to generate more biased data than whole-exome or whole-genome sequencing studies. Additionally, we did not attempt to validate all rare variants identified by alternative methods, but rather performed it only for selected variants such as Sanger sequencing of rare nonsynonymous variants of *VSTM1* and TaqMan genotyping of 37 variants from 200 samples. Finally, we included approximately 2,000 subjects for sequencing, which might not be enough of a sample size to discover rare variants. Consequently, this lack of power may lead most of the analysis in the study, such as the single-variant association analysis, gene-based tests, and enrichment tests, to be less significant with weak association.

However, to our knowledge, this study represents the largest sequencing study that evaluated the largest number of candidate genes with the largest case controls for RA until now. Despite negative findings, further replication of the possible single variants or rare variants in the study will be of some interest.

## Conclusions

We were unable to identify rare coding variants with large effect of 398 targeted genes. Despite much anticipation regarding missing heritability, our study raises skepticism about next-generation sequencing of targeted genes in order to discover rare variants with large effect for complex traits like RA. With the advance of genetic technology such as capturing, sequencing of targeted genes, and whole-exome or genome sequencing with a lot of subjects could define more details about the genetic architecture of RA in future.

## Additional file

Additional file 1: Table S1. Targeted gene coverage rate (percentage) of coding variants sequenced within 398 genes via exon sequencing. **Table S2**. Quality metrics for exon sequencing of 398 targeted genes. **Table S3**. Concordance of targeted exon sequencing (NGS) with other datasets (GWAS, iCHIP), and validation data (Taqman). **Table S4**. Single-variant test: results for exon sequencing of nonsynonymous variants (*P* <0.01). **Table S5**. Meta-analysis of single-association tests for coding variants (NGS + GWAS + iCHIP). **Figure S1**. A multifaceted approach in selecting target genes for resequencing in RA. **Figure S2**. Targeted exon sequencing pipeline. **Figure S3**. Principle component analysis for targeted exon sequencing. **Figure S4**. High-quality variants identified by targeted exon sequencing. **Figure S5**. Gene-based analysis of rare nonsynonymous variants in RA.

## Abbreviations

Chr: chromosome
db: database
GWAS: genome-wide association studies
HLA: human leukocyte antigen
HWE: Hardy-Weinberg equilibrium
iCHIP: immunochip
LD: linkage disequilibrium
MAF: minor allele frequency
MHC: major histocompatibility complex
NGS: next-generation sequencing
OR: odds ratio
PC: principal component
RA: rheumatoid arthritis
SD: standard deviation
SEM: standard error of the mean
SNP: single nucleotide polymorphism
SNV: single nucleotide variant
