# Supplementary material for: Targeted exon sequencing fails to identify rare coding variants with large effect in rheumatoid arthritis
Source: Arthritis Res Ther. 2014 Sep 30;16(5):447. doi: 10.1186/s13075-014-0447-7 (PMC4203956; doi:10.1186/s13075-014-0447-7)

**Supplementary Data**

**Targeted exon sequencing fails to identify rare coding variants with large effect in rheumatoid arthritis**

So-Young Bang,^1^ Young-Ji Na,^1^ Kwangwoo Kim,^1^ Young Bin Joo,^1^ Youngho Park,^2^ Jaemoon Lee,^2^ Sun-Young Lee,^3^ Adnan A Ansari,^3^ Junghee Jung,^4^ Hwanseok Rhee,^4^ Jong-Young Lee,^5^ Bok-Ghee Han,^5^ Sung-Min Ahn,^3,6^ Sungho Won,^2^ Hye-Soon Lee,^1,*^ and Sang-Cheol Bae^1,*^

^1^Department of Rheumatology, Hanyang University Hospital for Rheumatic Diseases, Seoul 133-792, Republic of Korea; ^2^Department of Applied Statistics, Chung-Ang University, Seoul 156-755, Republic of Korea; ^3^Center for Cancer Genome Discovery, Asan Institute for Life Science, University of Ulsan college of Medicine, Asan Medical Center, Seoul 138-736, Republic of Korea; ^4^Bioinfomatics Center, Macrogen Inc 153-023, Republic of Korea; ^5^Center for Genome Science, Korea National Institute of Health, Osong Health Technology, Chungcheongbuk-do 363-700, Republic of Korea; ^6^Department of Bioinformatics, University of Ulsan College of Medicine, Asan Medical Center, Seoul 138-736, Republic of Korea

*Correspondence: lhberon@hanyang.ac.kr (H.S.L.**),** scbae@hanyang.ac.kr (S.C.B.)

Table S1. Targeted gene coverage rate (percentage) of coding variants sequenced within 398 genes via exon sequencing

| Target gene coverage, % | | | | | | | | | | | | | | | | | |
| --- | --- | --- | --- | --- | --- | --- | --- | --- | --- | --- | --- | --- | --- | --- | --- | --- | --- |
| Gene | db^*^ | Target^*^ | *Gene* | db | Target | Gene | db | Target | Gene | db | Target | Gene | db | Target | Gene | db | Target |
| *ABCB4* | 82.0 | 98.4 | *CPS1* | 79.7 | 99.9 | *GPRC5A* | 92.6 | 97.8 | *LYG1* | 96.3 | 96.3 | *POLR1A* | 81.7 | 83.1 | *SP140* | 80.7 | 90.6 |
| *ABCC1* | 74.2 | 76.1 | *CSMD1* | 88.6 | 92.8 | *GPX3* | 70.6 | 70.6 | *LYG2* | 77.6 | 99.1 | *PPARG* | 97.2 | 97.6 | *SP140L* | 74.0 | 92.8 |
| *ACCN5* | 80.0 | 100 | *CTLA4* | 80.1 | 95.2 | *GRIN2B* | 85.6 | 92.3 | *LZTFL1* | 92.0 | 100 | *PRDM1* | 88.9 | 95.7 | *SPEF2* | 84.4 | 98.9 |
| *ACTR2* | 85.1 | 95.2 | *CTNNA3* | 90.9 | 99.7 | *GRM1* | 78.0 | 84.2 | *MADD* | 85.8 | 90.1 | *PRDM4* | 83.2 | 98.0 | *SPIC* | 96.7 | 99.9 |
| *ADAM18* | 77.2 | 99.9 | *CXCR5* | 78.2 | 78.2 | *GSDMB* | 93.3 | 96.3 | *MAGI3* | 77.9 | 90.8 | *PRDM9* | 98.3 | 98.3 | *SPOCK1* | 76.1 | 78.7 |
| *ADAM23* | 83.2 | 88.1 | *CYTH4* | 71.5 | 71.5 | *GSDMC* | 83.8 | 93.2 | *MAP2* | 81.6 | 96.5 | *PRKCB* | 73.5 | 87.5 | *SPP2* | 88.0 | 96.6 |
| *ADAM28* | 95.0 | 100 | *DAP* | 83.5 | 90.2 | *GUCY1B3* | 91.8 | 99.6 | *MAP3K8* | 90.9 | 91.7 | *PRKCE* | 77.1 | 82.3 | *SPRED2* | 82.7 | 82.7 |
| *ADAMTS12* | 83.1 | 89.5 | *DBC1* | 88.7 | 93.7 | *GYS2* | 91.0 | 100 | *MAPK14* | 88.4 | 99.3 | *PRKCH* | 84.3 | 92.8 | *SSR2* | 98.8 | 98.8 |
| *AHSA2* | 100 | 100 | *DCHS2* | 77.1 | 81.6 | *HERC6* | 77.7 | 86.4 | *MAPK8* | 77.6 | 99.6 | *PRKCQ* | 86.8 | 96.7 | *ST7L* | 73.6 | 85.7 |
| *AKAP11* | 90.7 | 100 | *DDB2* | 90.7 | 90.7 | *HHAT* | 78.8 | 83.9 | *MATN2* | 89.9 | 89.8 | *PROM1* | 83.8 | 93.9 | *STAT1* | 82.4 | 98.5 |
| *AKAP13* | 85.2 | 92.4 | *DDC* | 92.4 | 96.1 | *HIF1A* | 74.4 | 98.0 | *MBD4* | 94.1 | 93.1 | *PSMA6* | 99.8 | 100 | *STAT4* | 70.0 | 100 |
| *AKAP2* | 80.0 | 87.8 | *DDR2* | 95.6 | 96.3 | *HKR1* | 88.9 | 99.9 | *MBOAT1* | 89.9 | 93.9 | *PTCD3* | 70.9 | 97.5 | *STIM1* | 73.4 | 73.4 |
| *ALDH1A3* | 74.8 | 76.0 | *DDX6* | 90.5 | 100 | *ICA1* | 83.1 | 98.8 | *MCM7* | 78.4 | 80.0 | *PTPN11* | 80.0 | 94.2 | *SULT1E1* | 79.5 | 100 |
| *ALMS1* | 93.8 | 98.2 | *DGKB* | 75.0 | 100 | *IFI16* | 77.1 | 99.1 | *MCOLN2* | 87.8 | 100 | *PTPN22* | 83.4 | 100 | *SUSD1* | 88.8 | 98.6 |
| *ALPK2* | 91.4 | 99.6 | *DGUOK* | 83.6 | 83.6 | *IFT88* | 77.7 | 100 | *MED1* | 82.3 | 99.5 | *PTPRA* | 85.0 | 93.7 | *SYNPR* | 93.9 | 97.7 |
| *AMPD3* | 74.3 | 74.1 | *DLD* | 75.0 | 100 | *IKZF3* | 99.3 | 99.3 | *METTL4* | 95.0 | 100 | *PTPRC* | 70.0 | 100 | *TACR1* | 81.0 | 81.0 |
| *ANKRD55* | 92.4 | 92.4 | *DNAH5* | 92.3 | 97.5 | *IL12RB2* | 96.6 | 97.4 | *MLL* | 85.5 | 96.2 | *PTPRD* | 92.6 | 98.9 | *TAF11* | 99.9 | 100 |
| *ANXA13* | 85.2 | 86.3 | *DNAJB11* | 85.0 | 98.8 | *IL18RAP* | 97.9 | 97.9 | *MMP16* | 99.6 | 99.6 | *PTPRG* | 80.3 | 89.9 | *TAGAP* | 73.0 | 77.1 |
| *APBB2* | 95.3 | 95.3 | *DPY19L3* | 77.8 | 99.2 | *IL1R1* | 98.2 | 100 | *MMP26* | 75.4 | 99.2 | *PTPRH* | 73.7 | 73.6 | *TBC1D1* | 85.6 | 86.9 |
| *ARHGAP15* | 89.7 | 100 | *DRG2* | 84.5 | 84.5 | *IL2* | 100 | 100 | *MPPED2* | 99.0 | 99.0 | *PUS10* | 76.1 | 98.8 | *TBL1XR1* | 75.2 | 100 |
| *ARHGAP26* | 85.4 | 90.4 | *DTNA* | 90.2 | 95.6 | *IL21* | 89.4 | 100 | *MTRR* | 90.8 | 98.4 | *PXK* | 77.5 | 90.5 | *TBP* | 87.8 | 99.1 |
| *ARHGEF3* | 89.1 | 91.8 | *EBF1* | 76.8 | 77.6 | *IL23R* | 85.3 | 100 | *MYO10* | 80.5 | 85.5 | *PYGL* | 88.5 | 90.7 | *TBPL2* | 83.7 | 89.9 |
| *ARID5B* | 88.4 | 93.0 | *EFR3A* | 85.1 | 99.0 | *IL2RA* | 88.8 | 91.3 | *MYO9A* | 80.4 | 98.3 | *R3HDM2* | 73.0 | 75.2 | *TCP11* | 89.5 | 89.4 |
| *ASB3* | 80.0 | 100 | *EGFR* | 84.2 | 85.1 | *IL6R* | 81.3 | 81.3 | *MYOM2* | 79.3 | 81.7 | *RAB23* | 88.0 | 100 | *TEAD1* | 96.7 | 96.9 |
| *ATG16L1* | 89.6 | 92.7 | *ELAVL1* | 95.8 | 95.8 | *IL6ST* | 85.8 | 100 | *NAB1* | 93.2 | 97.0 | *RAC1* | 80.3 | 87.3 | *TG* | 84.5 | 85.2 |
| *ATP2B4* | 92.4 | 96.4 | *ELMO1* | 93.4 | 93.4 | *IL7R* | 78.8 | 99.7 | *NCALD* | 95.2 | 93.9 | *RAG1* | 94.8 | 99.4 | *TGFBR3* | 81.0 | 84.2 |
| *ATP8B2* | 76.2 | 78.2 | *ENOX1* | 86.5 | 92.8 | *ILKAP* | 71.1 | 71.2 | *NCF2* | 87.0 | 90.8 | *RASGRP1* | 96.7 | 96.8 | *THEMIS* | 83.8 | 100 |
| *BACH2* | 84.2 | 86.3 | *ENPP6* | 90.5 | 90.5 | *IPMK* | 72.5 | 84.0 | *NEDD4L* | 85.6 | 96.5 | *RBM17* | 88.7 | 96.4 | *TLR10* | 89.6 | 100 |
| *BAZ1A* | 74.8 | 99.7 | *ENSA* | 84.3 | 94.0 | *ITGAL* | 79.8 | 86.8 | *NEK2* | 95.3 | 94.7 | *RBPJ* | 77.4 | 94.1 | *TMEM174* | 98.0 | 98.0 |
| *BBS9* | 85.9 | 99.9 | *ERAP1* | 94.7 | 98.3 | *ITGB6* | 86.7 | 90.9 | *NETO1* | 92.7 | 98.6 | *REV3L* | 89.1 | 100 | *TNC* | 91.5 | 91.8 |
| *BCAS1* | 93.3 | 95.4 | *ERCC8* | 92.2 | 99.1 | *ITIH2* | 82.4 | 98.3 | *NLGN4X* | 87.0 | 89.0 | *RGL1* | 89.2 | 97.0 | *TNFAIP3* | 70.4 | 80.1 |
| *BEST3* | 73.3 | 90.0 | *ERRFI1* | 97.8 | 99.5 | *JAK2* | 82.9 | 100 | *NLRP4* | 90.7 | 95.9 | *RIMS1* | 71.7 | 80.2 | *TNFRSF9* | 70.0 | 100 |
| *C11orf30* | 88.1 | 99.6 | *ETS1* | 90.4 | 90.3 | *KAL1* | 86.2 | 90.2 | *NMI* | 82.0 | 100 | *RNASET2* | 72.6 | 87.2 | *TNFSF15* | 83.6 | 83.6 |
| *C1orf68* | 92.2 | 92.2 | *ETV5* | 82.4 | 90.3 | *KALRN* | 80.2 | 85.7 | *NMNAT2* | 79.2 | 79.2 | *RNF114* | 83.0 | 83.0 | *TNFSF4* | 86.9 | 95.4 |
| *C3* | 72.0 | 74.0 | *EXT1* | 93.3 | 96.6 | *KCNH7* | 99.3 | 100 | *NRXN1* | 82.5 | 84.7 | *ROBO2* | 84.9 | 98.5 | *TNN* | 76.5 | 79.8 |
| *C5* | 85.0 | 100 | *EYA1* | 80.4 | 95.6 | *KCNQ5* | 76.3 | 84.4 | *NRXN3* | 90.7 | 97.4 | *RORA* | 79.4 | 89.2 | *TNPO3* | 98.0 | 98.0 |
| *C6orf105* | 100 | 100 | *EYA2* | 73.3 | 83.0 | *KDR* | 86.5 | 97.4 | *NXPH1* | 97.1 | 100 | *RPL3* | 77.9 | 80.7 | *TRAF3IP2* | 86.8 | 86.8 |
| *C6orf99* | 98.4 | 98.4 | *EYA4* | 81.1 | 96.5 | *KIAA0391* | 92.0 | 100 | *ODZ4* | 76.3 | 77.6 | *RRAGC* | 79.9 | 99.9 | *TRAF6* | 90.0 | 100 |
| *CA8* | 88.4 | 92.4 | *FAM69A* | 95.3 | 95.0 | *KIAA1109* | 90.9 | 100 | *OLFM4* | 95.2 | 97.4 | *RSPH1* | 92.0 | 97.6 | *TRIM9* | 73.5 | 73.5 |
| *CAPN9* | 74.7 | 77.5 | *FAM83D* | 70.7 | 72.0 | *KIAA1841* | 75.0 | 100 | *OR51A7* | 100 | 100 | *RSPH3* | 78.6 | 78.7 | *TRPM8* | 94.0 | 96.5 |
| *CAST* | 79.8 | 98.0 | *FAP* | 77.0 | 100 | *KIAA1967* | 71.0 | 71.0 | *OR6C1* | 82.6 | 96.4 | *RYR2* | 87.2 | 98.3 | *TSC1* | 81.0 | 93.5 |
| *CCNYL1* | 75.2 | 79.2 | *FARP1* | 72.8 | 76.7 | *KIF23* | 85.0 | 100 | *OR6C6* | 90.5 | 100 | *RYR3* | 88.2 | 93.0 | *TUBAL3* | 94.9 | 94.9 |
| *CCR4* | 100 | 100 | *FBXL20* | 92.0 | 96.9 | *KIF5A* | 78.6 | 82.6 | *OS9* | 73.4 | 82.7 | *SCFD2* | 79.5 | 79.0 | *UBASH3A* | 78.5 | 78.4 |
| *CCR6* | 100 | 100 | *FBXO34* | 98.0 | 98.9 | *KIN* | 95.1 | 99.5 | *PALM2* | 77.3 | 94.1 | *SCN7A* | 81.9 | 100 | *UBE2L3* | 97.0 | 97.0 |
| *CCR7* | 91.0 | 91.0 | *FCAMR* | 80.0 | 79.9 | *KIT* | 98.4 | 98.6 | *PAPOLG* | 75.1 | 99.1 | *SCUBE3* | 72.6 | 72.5 | *UGT3A1* | 94.0 | 94.0 |
| *CCRL2* | 97.9 | 99.9 | *FCGR2A* | 95.7 | 99.7 | *KLF12* | 93.7 | 96.6 | *PAPSS1* | 96.2 | 96.1 | *SERBP1* | 80.0 | 90.3 | *UGT3A2* | 86.2 | 92.5 |
| *CCRN4L* | 83.5 | 84.4 | *FCRL3* | 90.9 | 97.8 | *KLF3* | 91.8 | 91.4 | *PAQR5* | 78.7 | 89.6 | *SGCZ* | 97.1 | 97.1 | *UHRF1BP1* | 94.1 | 95.0 |
| *CD2* | 85.1 | 95.3 | *FCRLA* | 74.2 | 83.5 | *KLHL8* | 99.6 | 100 | *PARD3* | 78.2 | 91.1 | *SGIP1* | 82.1 | 99.0 | *UNC13C* | 88.9 | 100 |
| *CD244* | 89.8 | 95.1 | *FGFR1OP* | 84.8 | 91.0 | *KLRB1* | 83.0 | 100 | *PARK7* | 75.6 | 99.5 | *SGMS1* | 89.5 | 89.5 | *UNC80* | 89.0 | 94.2 |
| *CD40* | 71.3 | 71.2 | *FGFR2* | 85.1 | 97.6 | *KPRP* | 90.1 | 90.5 | *PBX3* | 95.6 | 99.2 | *SIAE* | 96.1 | 96.1 | *USP34* | 72.7 | 99.7 |
| *CD44* | 90.4 | 96.2 | *FLI1* | 78.5 | 87.2 | *KRT13* | 73.5 | 74.0 | *PCDH11X* | 96.4 | 99.6 | *SIGLEC6* | 71.6 | 73.8 | *USP37* | 79.8 | 100 |
| *CD80* | 93.7 | 100 | *FLT1* | 91.6 | 95.8 | *KRT24* | 87.2 | 94.4 | *PCLO* | 91.3 | 97.3 | *SIRPG* | 78.6 | 78.6 | *VSTM1* | 89.3 | 93.7 |
| *CD86* | 90.0 | 100 | *FMNL3* | 73.8 | 79.3 | *KRT25* | 100 | 100 | *PDE4D* | 76.8 | 83.7 | *SLC16A7* | 80.8 | 100 | *WDFY4* | 78.8 | 79.8 |
| *CDC14A* | 95.3 | 99.3 | *FOXP1* | 79.1 | 98.2 | *KRT27* | 95.2 | 95.3 | *PDGFD* | 89.0 | 100 | *SLC22A2* | 80.9 | 80.7 | *WDR7* | 86.7 | 94.0 |
| *CDK12* | 88.6 | 97.7 | *FREM1* | 93.7 | 99.1 | *KY* | 76.2 | 76.1 | *PDSS2* | 85.0 | 84.8 | *SLC22A4* | 76.3 | 80.1 | *WWOX* | 84.4 | 91.4 |
| *CDK4* | 87.4 | 87.7 | *FRMD4B* | 90.4 | 94.7 | *LAMB1* | 84.6 | 86.3 | *PEBP4* | 88.1 | 88.1 | *SLC24A3* | 85.1 | 87.9 | *XCL1* | 94.5 | 94.5 |
| *CETP* | 76.2 | 83.0 | *FRMD6* | 91.9 | 97.7 | *LAMP3* | 96.0 | 96.0 | *PER3* | 86.5 | 92.1 | *SLC25A12* | 83.3 | 99.3 | *XPO1* | 87.9 | 100 |
| *CHST9* | 99.7 | 100 | *FSHR* | 96.2 | 96.4 | *LCP2* | 92.4 | 93.4 | *PHLPP1* | 72.0 | 82.5 | *SLC25A5* | 89.6 | 89.6 | *YARS2* | 73.2 | 72.2 |
| *CIT* | 84.2 | 89.3 | *FSTL4* | 72.9 | 80.7 | *LDHB* | 83.0 | 100 | *PHTF1* | 71.9 | 94.8 | *SLC26A3* | 94.8 | 100 | *YPEL5* | 100 | 100 |
| *CLCA2* | 93.7 | 99.9 | *GALNTL6* | 72.1 | 88.1 | *LEPREL1* | 76.4 | 82.2 | *PIGR* | 70.9 | 70.9 | *SLC35D1* | 71.2 | 84.5 | *ZC3H8* | 83.0 | 99.9 |
| *CNRIP1* | 72.0 | 72.0 | *GCA* | 79.6 | 96.6 | *LGALS2* | 96.7 | 97.1 | *PIP4K2C* | 77.7 | 87.3 | *SLC44A2* | 82.4 | 83.6 | *ZEB1* | 84.2 | 100 |
| *CNTN4* | 91.8 | 99.9 | *GCG* | 100 | 100 | *LGALSL* | 93.0 | 93.0 | *PKP2* | 72.4 | 75.4 | *SLC9A8* | 73.5 | 78.3 | *ZNF365* | 82.8 | 96.8 |
| *CNTN6* | 85.0 | 100 | *GLDC* | 81.2 | 95.4 | *LHX9* | 75.5 | 76.1 | *PLCL2* | 90.7 | 89.0 | *SMAD2* | 88.8 | 100 | *ZNF423* | 72.9 | 72.9 |
| *COL28A1* | 88.4 | 97.9 | *GLIS3* | 79.4 | 82.7 | *LMOD3* | 92.2 | 100 | *PLEK* | 98.8 | 98.8 | *SMG7* | 91.9 | 96.6 | *ZNF451* | 87.6 | 97.0 |
| *CPEB2* | 71.0 | 100 | *GLT8D2* | 97.8 | 99.8 | *LPAR1* | 89.1 | 88.8 | *PLSCR2* | 89.3 | 90.9 | *SNAP25* | 77.4 | 91.8 | *ZNF804A* | 97.3 | 100 |
| *CPEB4* | 87.8 | 99.9 | *GPR137B* | 83.0 | 89.5 | *LRRTM3* | 96.5 | 97.2 | *PLSCR4* | 92.5 | 97.5 | *SNRPC* | 99.1 | 99.5 | *ZPBP2* | 71.5 | 91.2 |
|  |  |  | *GPR65* | 90.9 | 100 |  |  |  |  |  |  | *SNTB1* | 75.5 | 82.0 |  |  |  |

^*^Percentage of coding sites sequenced with > 20× coverage and quality score > 30 in at least 80% of cases and controls in the public database (db) or in the designed targeting genes

Table S2. Quality metrics for exon sequencing of 398 targeted genes.

|  | Exon Sequencing | |
| --- | --- | --- |
|  | RA cases (n=1,217) | Controls (n =717) |
| Ti/Tv  (mean ± SD) | 2.93 ± 0.1 | 2.92 ± 0.1 |
| Sequencing gene/target gene (%) | 99.7% | 99.6% |
| Mean depth | 186.5 | 188.8 |
| Target site more than 20× coverage (%) | 92.3% | 91.6% |
| Target genes | 398 | |
| SNVs (minimal depth >20×, quality score >30, Call Rate >90%, Control HWE >0.01) | 10,588 | |
| Rare SNVs (minimal depth >20×, quality score >30, Call Rate >90%, Control HWE >0.01, MAF <5%) | 9,597 | |

^*^The following abbreviations are used: RA, rheumatoid arthritis; Ti/Tv, transition/transversion; SNV, single nucleotide variant; HWE, Hardy-Weinberg equilibrium; MAF, minor allele frequency.

Table S3. Concordance of targeted exon sequencing (NGS) with other datasets (GWAS, iCHIP), and validation data (Taqman).

|  |  | |  | | Concordance | | | |
| --- | --- | --- | --- | --- | --- | --- | --- | --- |
|  | Sample No. | SNV No. | | Overall genotype concordance | | Non-reference sensitivity | | Non-reference discrepancy rate |
| GWAS vs NGS (total) | 226 | 385 | | 98.4% | | 97.9% | | 2.4% |
| GWAS vs NGS (MAF<5%) |  | 73 | | 99.7% | | 98.8% | | 0.8% |
| Immunochip vs NGS (total) | 163 | 377 | | 98.2% | | 97.6% | | 3.5% |
| Immunochip vs NGS (MAF<5%) |  | 152 | | 99.3% | | 95.5% | | 4.5% |
| Validation (Taqman) | 200 | 37 | | 98.3% | | 97.9% | | 5.1% |
| Validation (Taqman) (MAF<5%) |  | 13 | | 99.8% | | | 99.4% | 0.1% |

^*^The following abbreviations are used: No, number; GWAS, genome-wide association studies; NGS, next generation sequencing; SNV, single nucleotide variant; MAF, minor allele frequency.

Table S4. Single variant test: Results for exon sequencing of nonsynonymous variants (p <0.01).

| Gene | Chr | Position | EA/NEA | function | case.MAF | control.MAF | p-value^*^  Fisher’s exact test | p-value^†^  PC adjusted logistic-regression | OR |
| --- | --- | --- | --- | --- | --- | --- | --- | --- | --- |
| *TGFBR3* | 1 | 92177829 | T/A | nonsynonymous | 0 | 0.00418 | 0.00258 | - | - |
| *ALMS1* | 2 | 73716810 | G/A | nonsynonymous | 0.27855 | 0.23570 | 0.00358 | 0.00637 | 1.24 |
| *ALMS1* | 2 | 73717103 | G/C | nonsynonymous | 0.27979 | 0.23710 | 0.00364 | 0.00637 | 1.24 |
| *MYOM2* | 8 | 2048831 | A/G | nonsynonymous | 0.36796 | 0.32163 | 0.00367 | 0.00528 | 1.23 |
| *CSMD1* | 8 | 3076959 | T/C | nonsynonymous | 0.06286 | 0.04114 | 0.00425 | 0.00590 | 1.56 |
| *SPP2* | 2 | 234959637 | T/A | nonsynonymous | 0.11463 | 0.08577 | 0.00453 | 0.00575 | 1.38 |
| *RYR3* | 15 | 33927988 | G/A | nonsynonymous | 0.00043 | 0.00507 | 0.00518 | - | 0.09 |
| *LGALS2* | 22 | 37966314 | C/T | nonsynonymous | 0.05124 | 0.03210 | 0.00542 | 0.00673 | 1.63 |
| *LRRTM3* | 10 | 68857397 | T/G | nonsynonymous | 0.13476 | 0.16806 | 0.00553 | 0.00745 | 0.78 |
| *FBXO34* | 14 | 55818411 | A/T | nonsynonymous | 0 | 0.00349 | 0.00697 | - | - |
| *REV3L* | 6 | 111695603 | G/A | nonsynonymous | 0.00082 | 0.00558 | 0.00718 | - | 0.13 |
| *TNFAIP3* | 6 | 138196066 | T/G | nonsynonymous | 0.06697 | 0.04603 | 0.00734 | 0.00714 | 1.50 |
| *ALMS1* | 2 | 73675669 | T/G | nonsynonymous | 0.27426 | 0.23570 | 0.00880 | 0.01501 | 1.21 |

The following abbreviations are used: Chr, chromosome; EA, effect allele; NEA, non-effect allele; MAF, minor allele frequency; OR, odds ratio.

^*^ P-value was calculated using Fisher’s exact tests in common, low frequency, and rare variants.

^†^ P-value was calculated by principal-component (PC) adjusted logistic-regression in common and low frequency variants (MAF>1%).

Table S5. Meta-analysis of single association tests for coding variants [NGS+GWAS + iCHIP].

| Gene | Chr -position |  | rs_Number | NGS (I) | | | GWAS (II) | iCHIP (III) | Meta P (I+II+III) |
| --- | --- | --- | --- | --- | --- | --- | --- | --- | --- |
|  |  |  |  | case MAF | control MAF | P | P | P |  |
| *PRKCH* | 14-6997226 | synonymous | rs1088680 | 0.3223 | 0.2890 | 0.0421 | 0.0128 | 0.0024 | 3.16E-05 |
| *GLT8D2* | 12-104408794 | nonsynonymous | rs17035120 | 0.3484 | 0.3138 | 0.0320 | 0.0057 | 0.0162 | 1.20E-04 |
| *FCRL3* | 1-157668390 | nonsynonymous | rs7522061 | 0.4277 | 0.3952 | 0.0628 | 0.1150 | 0.0165 | 0.0020 |
| *CCR7* | 17-38711222 | synonymous | rs2229095 | 0.0222 | 0.0377 | 0.0026 | 0.1818 | 0.1064 | 0.0099 |
| *EBF1* | 5-158204425 | synonymous | rs1368298 | 0.3361 | 0.2992 | 0.0130 | 0.6791 | 0.1326 | 0.0851 |
| *IL12RB2* | 1-67861520 | synonymous | rs2229546 | 0.3885 | 0.3566 | 0.0446 | 0.2901 | 0.5548 | 0.2147 |

^*^The following abbreviations are used: GWAS, genome-wide association studies; NGS, next generation sequencing; Chr, chromosome; MAF, minor allele frequency.

Figure S1. A multifaceted approach in selecting target genes for resequencing in RA.

Of the non-MHC candidate genes, we included (a) 106 known RA risk loci identified via thorough literature review from previous case-control association studies, (b) 519 genes having SNPs with p<1×10^−3^ in our Korean iChip dataset (unpublished data), (c) 155 genes from 363 SNPs associated with risk of both RA and systemic lupus erythematosus (SLE) in our previous GWAS datasets, (d) 18 genes involved in RA-related pathways according to the i-GSEA4GWAS web server, (e) 65 genes identified by text-mining using GRAIL from recent GWAS data, (f) and 8 human homologues of mouse genes known to induce an RA-like phenotype from the Mouse Genome Database (MGD). We selected 398 genes with coding variants (>20× coverage and quality score >30) that covered more than 80% of the db coding-region annotated in more than 80% of sequenced subjects among 666 genes**.** The 398 genes included 54 known RA risk loci selected via thorough literature review from previous RA case-control association studies and 326 genes selected from GWAS or iChip dataset from Korean population (p < 1×10^−3^).


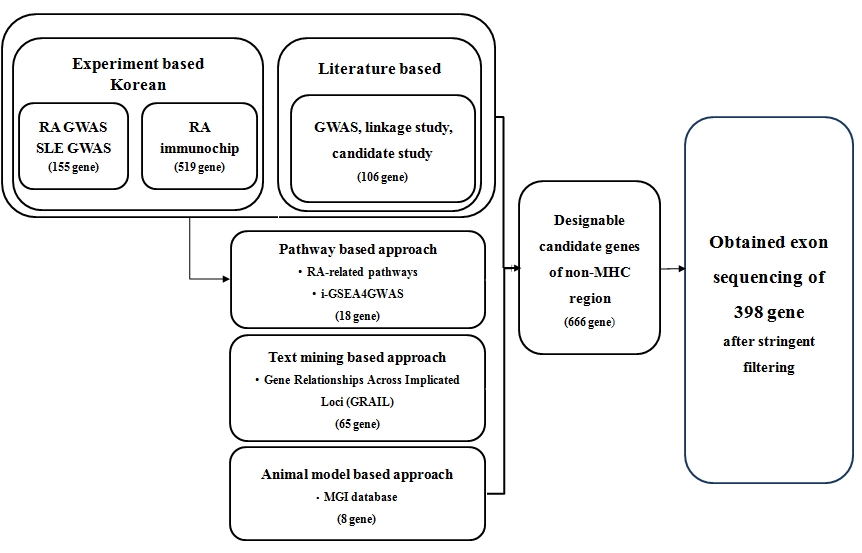


Figure S2. Targeted exon sequencing pipeline.

We enriched the target exons with Agilent’s SureSelect capture kit (target region=1.36 Mb) and SAM file that includes only reads that uniquely mapped to the reference genome, transforming it into BAM file with Samtools, and filtering out any reads not across the target regions, the information of which is informed by the manufacturer of SureSelect target enrichment system. This filtering is executed with the program named BED tools (version 2.15.0). The variant call files for total of 1,997 samples were generated through the targeted sequencing analysis pipeline.


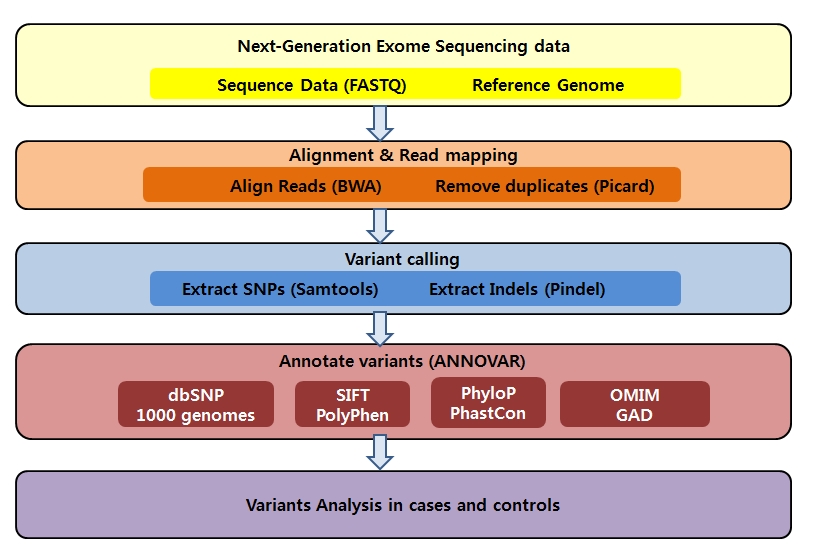


Figure S3. Principle component analysis for targeted exon sequencing.

Results of the principle component analysis for 1,252 RA cases and 745 controls.


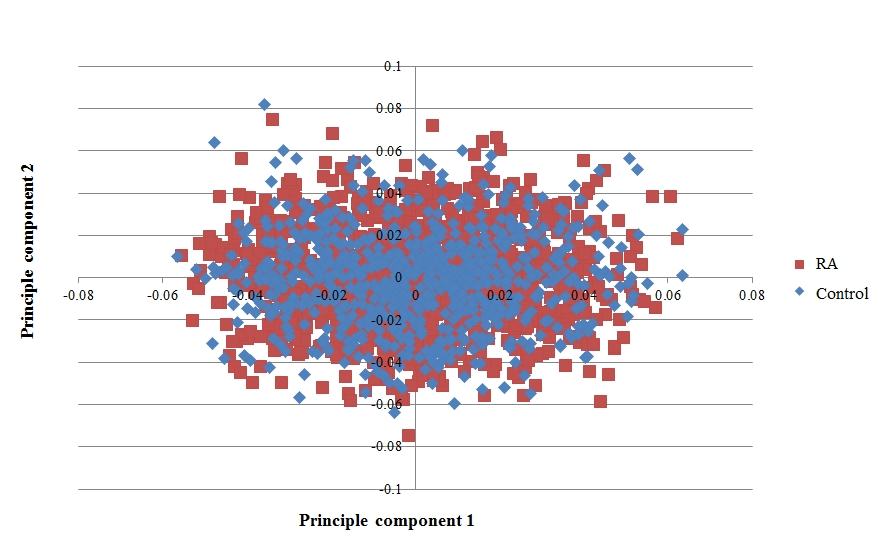


Figure S4. High quality variants identified by targeted exon sequencing.

We identified 9,597 high quality rare variants and 6,605 novel single-nucleotide variants (SNVs) that are not found in the dbSNP138 or 1000 genome project data sets using ESP6500 (http://evs.gs.washington.edu/EVS/) and ANOVAR among 1,217 RA cases and 717 healthy controls.


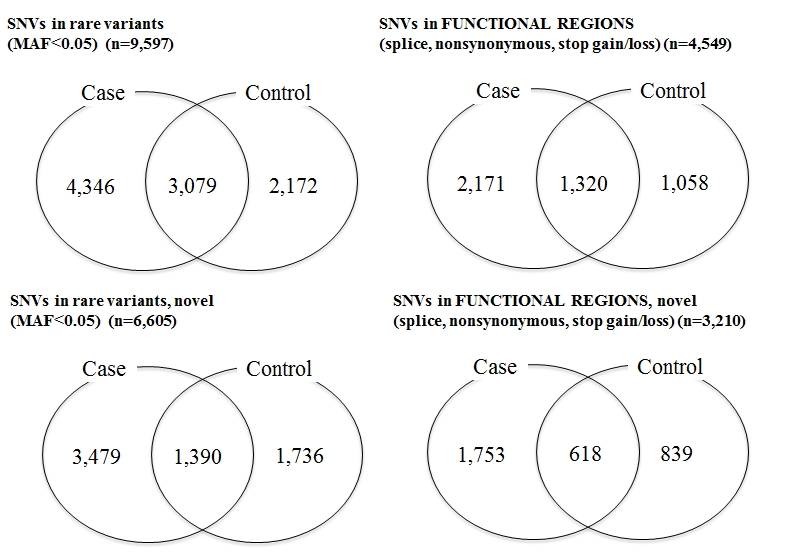


Figure S5. Gene-based analysis of rare nonsynonymous variants in RA

In a gene-based analysis of rare coding variant data (MAF < 5%), we considered both non-burden testing [optimal sequence kernel association test (SKAT-O)] and burden testing [SCORE-seq]. We also performed weighted tests with SIFT, PolyPhen, and CAROL scores to determine the functional effects of the nonsynonymous variants.


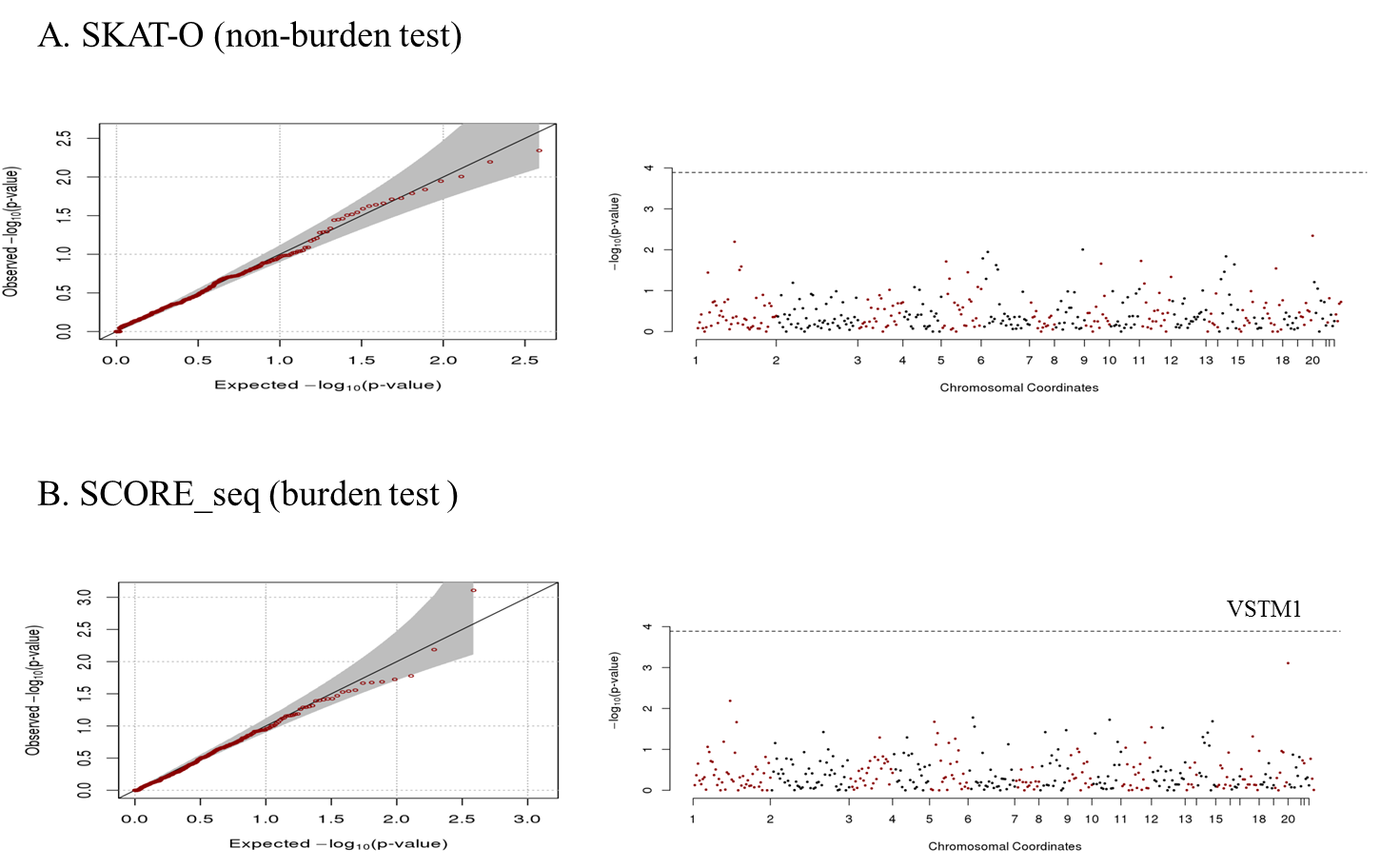


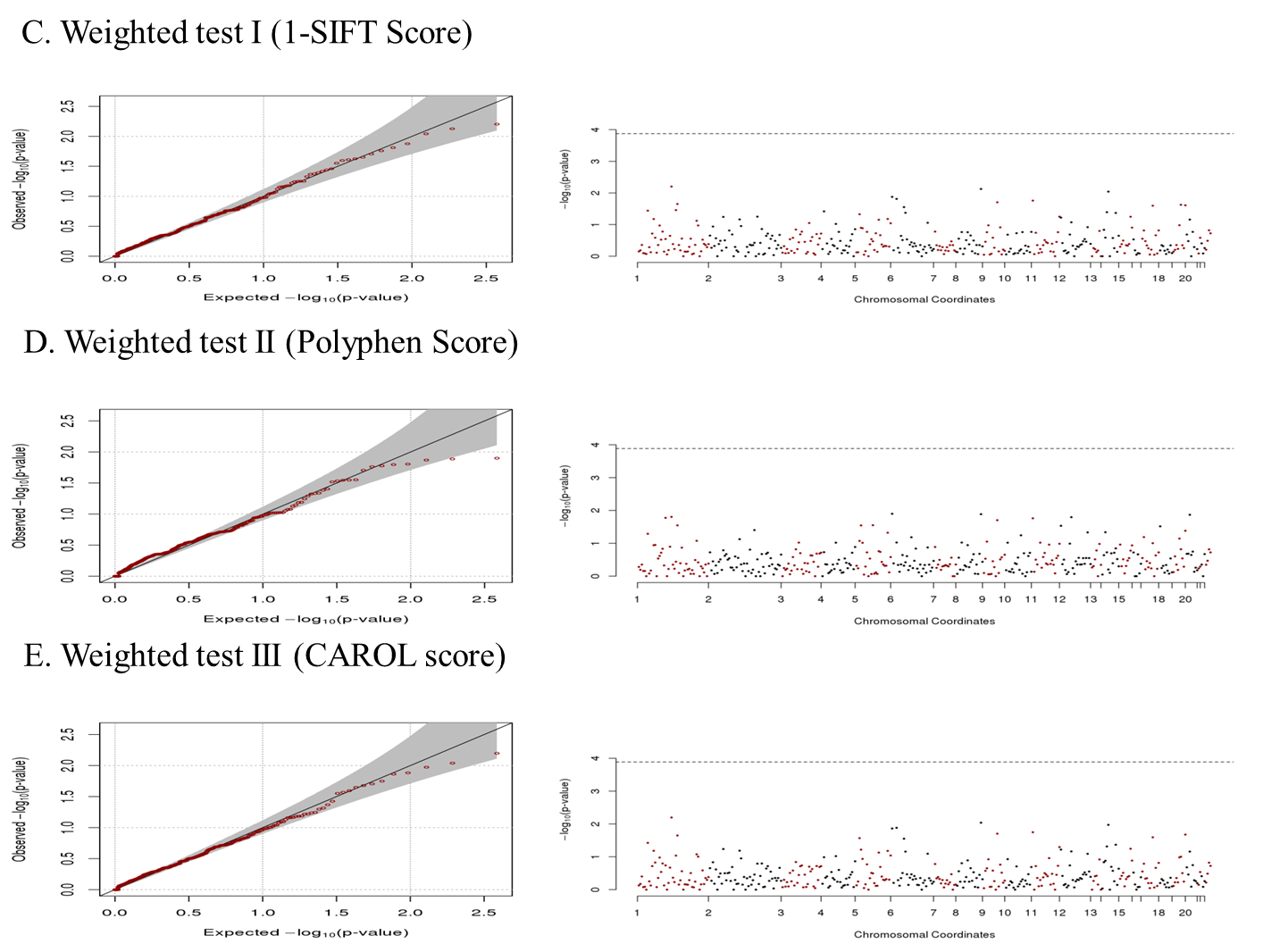

Supplement: Additional file 1: Table S1. — Targeted gene coverage rate (percentage) of coding variants sequenced within 398 genes via exon sequencing. Table S2. Quality metrics for exon sequencing of 398 targeted genes. Table S3. Concordance of targeted exon sequencing (NGS) with other datasets (GWAS, iCHIP), and validation data (Taqman). Table S4. Single-variant test: results for exon sequencing of nonsynonymous variants (P <0.01). Table S5. Meta-analysis of single-association tests for coding variants (NGS + GWAS + iCHIP). Figure S1. A multifaceted approach in selecting target genes for resequencing in RA. Figure S2. Targeted exon sequencing pipeline. Figure S3. Principle component analysis for targeted exon sequencing. Figure S4. High-quality variants identified by targeted exon sequencing. Figure S5. Gene-based analysis of rare nonsynonymous variants in RA. [file 13075_2014_447_MOESM1_ESM.doc]
